# Word imageability influences the emotionality effect in episodic memory

**DOI:** 10.1007/s10339-022-01102-4

**Published:** 2022-07-20

**Authors:** Claire Ballot, Christelle Robert, Stéphanie Mathey

**Affiliations:** 1 University of Bordeaux, Bordeaux, France; 2 Present Address: Faculty of Psychology and Educational Science, University of Geneva, Boulevard du Pont-d’Arve 40, 1211 Geneva 4, Switzerland

**Keywords:** Word imageability, Emotional words, Episodic memory, Free recall, Recognition

## Abstract

This study examines how and to what extent imageability influences the effect of word emotionality in episodic memory. A total of 52 young adults successively performed a free recall task and a recognition task in which word emotionality and imageability were orthogonally manipulated across six conditions of French words: low-imageability positive words (e.g., éloge [praise]), low-imageability negative words (e.g., viral [viral]), low-imageability neutral words (e.g., global [global]), high-imageability positive words (e.g., ourson [teddy]), high-imageability negative words (e.g., tornade [tornado]), and low-imageability neutral words (e.g., noyau [core]). The results from both the recall and the recognition memory tasks show that word imageability enhances memory performance. Importantly, word imageability interacted with word emotionality in both tasks. Specifically, we found that the advantage of emotional over neutral words in episodic memory performance emerged for high-imageability words only, as did the advantage of positive over negative words. These results highlight the role of imageability in the mechanisms underlying emotional word episodic memory.

## Introduction

The mechanisms and factors underlying episodic memory for emotional words have interested researchers for decades. Emotional valence (i.e., the degree to which a word is pleasant or not) is one of the most widely studied factors in episodic word memory (see Hamann 2001 for a review; Lau et al. 2018). Typically, emotional words were remembered better than neutral words (e.g., Adelman and Estes 2013; see also Snefjella and Kuperman 2016 for the role of emotional context). However, the story is a little more complicated when the polarity of the valence in the effects of emotional words in episodic memory is taken into account. Indeed, while some authors have shown a facilitatory effect of emotional words (whether positive or negative) compared to neutral words (e.g., Fleming et al. 2003), others have shown a preference for positive over neutral words (e.g., Ferré 2003), and still others a preference for negative rather than positive words (e.g., Dewhurst and Parry 2000). One explanation for these apparently inconsistent findings might reflect the lack of control for other lexical factors that have been shown to influence word memory, such as imageability (e.g., see Kousta et al. 2009 for a similar rationale in the field of word processing). Word imageability refers to the ease with which a word evokes a mental image (e.g., Gonthier et al. 2009). It has been shown as one of the most predictive factors in episodic memory performance, with high-imageability words being better recalled and recognized than low-imageability words (e.g., Ballot et al. 2021; Cortese et al. 2014; Khanna and Cortese 2021). The Dual-Coding Theory (Paivio 1991) was an early account of this facilitatory effect of word imageability in memory performance. This theory assumes a dual representation for high-imageability words in both linguistic and imagistic systems, whereas low-imageability words are only represented in the linguistic system. As a consequence, high-imageability words are better memorized.

In short, some studies on episodic memory have shown an influence of word emotional valence (e.g., Fleming et al. 2003) and others of word imageability (e.g., Ballot et al. 2021). While an interaction between these two meaning-based word characteristics appears plausible, it remains unexplored in episodic memory. In the field of lexical processing, few studies have examined the interaction between emotional valence and word concreteness, a dimension highly correlated with imageability (Kanske and Kotz 2007; Yao et al. 2016). By measuring event-related potentials during lexical decision tasks, concreteness was found to modulate the effect of emotional words (i.e., positive or negative words) during the late elaborate processing stage (i.e., LPC) that would reflect the intervention of mental imagery processes and semantic integration (Kanske and Kotz 2007; Yao et al. 2016). While imageability and concreteness and have been viewed as interchangeable variables (e.g., Reilly and Kean 2007) due to their strong correlation (e.g., Desrochers and Thompson 2009), imageability has been recently shown to be a better predictor of word memory performance compared to concreteness, suggesting that the visual modality is the most important modality during word encoding and/or retrieval (Khanna and Cortese 2021). Moreover, imageability would better reflects the mental imagery activity that is widely used in word learning (e.g., Citron et al. 2014) and, as such, is thought to be the critical mechanism involved in the concreteness effect of emotional words (Kanske and Kotz 2007). In order to identify the mechanisms involved in episodic memory for emotional words, it thus appears important to clarify the role of word imageability on the memorization of emotional words. While a large body of studies has focused on the distinct effect of word emotionality and word imageability on memory, the combined effect of emotional valence and word imageability in episodic memory remains unknown to date.

The main purpose of the present study was to investigate how and to what extent word imageability influences the effect of word emotionality in episodic memory. Word emotionality was operationalized by varying emotional valence and arousal together, with neutral words being less valenced and lower in arousal than emotional words (see also Kanske and Kotz 2007), which reflects a natural situation since these two emotional factors are related by a quadratic relationship (e.g., Citron et al. 2014; Gobin et al. 2017). Based on previous studies (e.g., Cortese et al. 2014; Dewhurst and Parry 2000), we hypothesized facilitatory effects of word imageability and emotionality in free recall and recognition. More precisely, high-imageability words should be better recalled and recognized than low-imageability words, as it should also occur for emotional words compared with neutral words. More importantly, given the critical role of mental imagery in word memory (Khanna and Cortese 2021), we expected emotional effects to be more prominent for high-imageability words than for low-imageability words in episodic memory tasks.

## Method

### Participants

A total of 52 young adults (*M* = 20.93 years, SD = 2.50) took part in the experiment. They were all native speakers of French (or had learned to write in French in preparatory school) and reported to have normal or corrected-to-normal vision.

### Materials

The 120 stimulus words of 4 to 7 letters and 1 or 2 syllables were selected from the French emotionality database EMA (Gobin et al. 2017) which provides emotional valence and arousal ratings for a set of French words. Emotional valence was manipulated so that 40 words were positive (*M* = 1.56, SD = 0.66), 40 were negative (*M* = − 1.63, SD = 0.66), and 40 were neutral (*M* = 0.16, SD = 0.59), *p*s < 0.001. Imageability for each word was rated by at least 50 young adults who did not participate in the experiment (*M* = 20.8 years, SD = 2.28) on a 7-point scale (1 = “words hardly imageable; 7 = “words easily imageable”; see Desrochers and Thompson 2009). Half of the words were highly-imageable (*M* = 5.70, SD = 0.91) and the other half were low-imageable (*M* = 2.77, SD = 0.48), *p*s < 0.001. In sum, valence and imageability were manipulated across 6 word conditions: 20 low-imageability positive words (e.g., éloge [praise]; valence, *M* = 1.50, SD = 0.41; imageability, *M* = 2.78, SD = 0.42), 20 low-imageability negative words (e.g., viral [viral]; valence, *M* = − 1.63, SD = 0.28; imageability, *M* = 2.8, SD = 0.41), 20 low-imageability neutral words (e.g., global [global]; valence, *M* = 0.12, SD = 0.27; imageability, *M* = 2.75, SD = 0.60), 20 high-imageability positive words (e.g., ourson [teddy]; valence, *M* = 1.62, SD = 0.45; imageability, *M* = 5.71, SD = 0.92), 20 high-imageability negative words (e.g., tornade [tornado]; valence, *M* = − 1.64, SD = 0.36; imageability, *M* = 5.71, SD = 0.98) and 20 high-imageability neutral words (e.g., noyau [core]; valence, *M* = 0.21, SD = 0.33; imageability, *M* = 5.68, SD = 0.87). Arousal scores were matched between positive and negative words, *p*s > 0.10 and were higher for emotional than for neutral words, *p*s < 0.001. Other word characteristics were taken from the French lexical database Lexique 3.8 (New et al. 2007). The six word conditions were matched for word frequency (in occurrences per million), number of syllables, number of letters, number of orthographic neighbors, OLD-20, number of phonological neighbors and PLD-20 (all *p*s > 0.10). For the free recall task, four lists of 30 stimuli were drawn up so that each list contained 5 words per experimental condition. For the purpose of the recognition task, 120 French words were selected as distractors such that word imageability and emotional valence were manipulated across the same word conditions as the experimental words (*p*s < 0.001). In addition, the distractors were matched with the experimental words on lexical frequency, number of syllables, number of letters, word imageability, emotional valence, and arousal scores (*p*s > 0.10).

### Procedure

After providing written consent, the participants were tested individually in a quiet room and successively performed computerized free-recall and recognition tasks. For the study phase, the words from each list were presented one at a time. The participants were instructed to read the words aloud and memorize them. Each word appeared in lowercase in the center of a computer screen for 3000 ms, and was preceded by a 1000-ms fixation cross. At the end of the list, the participants had to count down for 30 s and then recall words without any recall order or time limit restrictions. Another list then appeared and the same procedure was followed. The word presentation was randomized for each list, and list appearance order was counterbalanced across participants to control for list order effects. Immediately after the recall of the fourth list, the participants performed a recognition task in which they had to decide whether each word appearing on the screen was “new” or “old” by pressing one of the two buttons on the computer keyboard. The words were preceded by a 1000-ms fixation cross and remained on the screen until the participant responded. The 120 words from the previous free-recall task and the 120 new words used as distractors appeared randomly for each participant.

## Results

Separate analyses of variance (ANOVAs) were performed on hits for recall, hits for recognition memory, false alarms, and on a sensitivity index (*d*ʹ) based on the hits and false alarms with word imageability (low vs. high) and emotional valence (positive, negative and neutral) as within factors. Planned quadratic contrast analyses were conducted to test the effect of emotional words as compared to neutral words for each imageability condition. Orthogonal linear contrasts were also conducted to test for a polarity valence effect (positive vs. negative) among the emotional words. The results are shown in Table 1.

**Table 1:** Mean percentages and standard errors (in brackets) of hits in free recall and hits, false alarms and *d*' in memory recognition by word condition

	Low-imageability words			High-imageability words		
	Positive	Negative	Neutral	Positive	Negative	Neutral
Free recall						
Hits	21.77 (1.9)	24.13 (1.7)	23.7 (1.5)	37.21 (1.8)	32.98 (1.9)	30.0 (2.1)
Recognition						
Hits	67.02 (2.0)	69.33 (2.0)	69.04 (2.1)	77.98 (2.0)	74.03 (2.2)	73.26 (2.1)
FA	12.79 (1.5)	12.60 (1.4)	8.65 (1.2)	8.85 (1.2)	12.21 (1.7)	14.04 (1.6)
* d*ʹ	1.73 (0.07)	1.80 (0.08)	2.02 (0.10)	2.32 (0.10)	2.04 (0.08)	1.90 (0.09)

*FA* false alarms

### Free recall task

A main effect of word imageability was found, *F*(1, 51) = 65.73, *p* < 0.001, *η*^2^*p* = 0.56, with high-imageability words being better recalled (*M* = 33.40%) than low-imageability words (*M* = 23.21%). The main effect of word emotionality was not significant, *F*(2, 102) = 1.67, *p* = 0.19. Importantly, a significant interaction was found between word imageability and emotionality, *F*(2, 102) = 8.30, *p* < 0.001, *η*^2^*p* = 0.14. For low-imageability words, neither the quadratic, *F* < 1, nor the linear contrast, *F*(1, 51) = 1.69, *p* = 0.20, was significant. For high-imageability words the quadratic contrast was significant, *F*(1, 51) = 9.67, *p* = 0.003, *η*^2^*p* = 0.16, indicating that positive (*M* = 37.21%) and negative words (*M* = 32.98%) were better recalled than neutral words (*M* = 30.0%). The linear contrast was also significant, *F*(1, 51) = 5.96, *p* = 0.02, *η*^2^*p* = 0.11, indicating that positive words were better recalled than negative ones.

### Recognition task

The hit analysis showed that the main effect of word imageability was significant, *F*(1, 51) = 33.54, *p* < 0.001, *η*^2^*p* = 0.40. High-imageability words were better recognized (*M* = 75.10%) than low-imageability words (*M* = 68.46%). There was no main effect of word emotionality, *F* < 1. Finally, the interaction between word imageability and emotionality was significant, *F*(2, 102) = 4.36, *p* = 0.015, *η*^2^*p* = 0.08. For low-imageability words, neither the quadratic, *F* < 1, nor the linear contrasts, *F*(1, 51) = 2.23, *p* = 0.14, were significant. For high-imageability words, the quadratic contrast was marginally significant, *F*(1, 51) = 2.99, *p* = 0.09, *η*^2^*p* = 0.06. Positive (*M* = 77.98%) and negative words (*M* = 74.03%) tended to be better recognized than neutral words (*M* = 73.26%). The significant linear contrast indicated that positive words were better recalled than negative words, *F*(1, 51) = 6.37, *p* = 0.015, *η*^2^*p* = 0.11.

In the false alarm analysis, neither the main effect of word imageability, *F* < 1, nor of word emotionality, *F*(2, 102) = 1.47, *p* = 0.23, were reliable. Importantly, the interaction between word emotionality and imageability was significant, *F*(2, 102) = 15.37, *p* < 0.001, *η*^2^*p* = 0.23. For low-imageability words, the effect of word emotionality was significant for the quadratic contrast, *F*(1, 51) = 12.61, *p* = 0.001, *η*^2^*p* = 0.20. Neutral words exhibited fewer false alarms (*M* = 8.65%) than either positive (*M* = 12.79%) or negative words (*M* = 12.60%). No effect was found for the linear contrast *F* < 1. For high-imageability words, the quadratic contrast was significant, *F*(1, 51) = 11.40, *p* < 0.001, *η*^2^*p* = 0.18, with positive (*M* = 8.85%) and negative words (*M* = 12.21%) exhibiting fewer false alarms than neutral words (*M* = 14.04%). The linear contrast was also significant, *F*(1, 51) = 8.04, *p* = 0.007, *η*^2^*p* = 0.14, indicating that positive words exhibited fewer false alarms than negative words.

The *d*ʹ analysis showed a main effect of word imageability, *F*(1, 51) = 24.81, *p* < 0.001, *η*^2^*p* = 0.33, with high-imageability words better discriminated (*M* = 2.09) than low-imageability words (*M* = 1.85). No main effect of word emotionality was found, *F*(2, 102) = 1.42, *p* = 0.24. Again, the interaction effect was significant, *F*(2, 102) = 15.37, *p* < 0.001, *η*^2^*p* = 0.23. For low-imageability words, the effect of emotionality was significant for the quadratic contrast, *F*(1, 51) = 8.66, *p* = 0.005, *η*^2^*p* = 0.14, with positive (*M* = 1.73) and negative (*M* = 1.80) words being less-well discriminated than neutral words (*M* = 2.02). No effect was found for the linear contrast, *F* < 1. For highly-imageable words, the quadratic contrast was significant, *F*(1, 51) = 14.69, *p* < 0.001, *η*^2^*p* = 0.22, with positive (*M* = 2.32) and negative words (*M* = 2.04) better discriminated than neutral words (*M* = 1.90). The linear contrast was also significant, *F*(1, 51) = 11.25, *p* = 0.002, *η*^2^*p* = 0.18, showing that positive words were better discriminated than negative ones.

## Discussion

This study investigated how and to what extent word imageability influences the effect of word emotionality in episodic memory. As expected, the main effect of word imageability was discerned in both free recall and recognition tasks. Although we found no reliable main effect of word emotionality, we showed that the emergence of this effect consistently depended on word imageability in both recall and recognition tasks. Importantly, we found that for highly-imageable words only, episodic memory performance was enhanced for emotional words compared to neutral ones, and for positive words compared to negative ones. These results are discussed with regard to the role of word imageability in emotional word memory.

In a first finding, we confirmed data from previous studies showing that word imageability enhances episodic memory performance (see e.g., Ballot et al. 2021; Cortese et al. 2014; Khanna and Cortese 2021). We found the facilitatory effect of word imageability in both free recall and recognition to be consistent with the Dual-Coding theory (e.g., Paivio 1991). Within this framework, high-imageability words benefit from dual coding in imagistic and linguistic systems, while the processing of low-imageability words only occurs in a purely linguistic system. Mental imagery appears therefore to be a key process in high-imageability word memory whether in free recall or in recognition (see also Cortese et al. 2014).

The most important finding here was the interaction between word imageability and word emotionality in both recall and recognition. One could have expected that low-imageable words would benefit more from word emotionality than did high-imageable words since these latter words are already highly memorable due to their linguistic and imagistic coding (Paivio 1991; see also Miller and Roodenrys (2009) for a similar rationale when considering word frequency and concreteness in serial recall). However, our data converge towards the reverse pattern such that memory for high-imageability words benefit more from word emotionality than memory for low-imageability words*.* This memory advantage for high-imageability emotional words was more prominent for positive words than for negative words when considering hits in both free recall and recognition. Moreover, a mirror effect was found in the recognition task, with fewer false alarms for high-imageability emotional words than for high-imageability neutral ones. High-imageability positive words also produced fewer false alarms than high- imageability negative words. This pattern of data, which is supported by the analysis of discrimination accuracy (i.e., *d*ʹ value), indicates that highly-imageable emotional words, especially positive ones, were discriminated more readily than highly-imageable neutral words. The new finding that word imageability influences the effect of word emotionality is in line with previous studies conducted on the role of concreteness in visual lexical processing (e.g., Kanske and Kotz 2007; Yao et al. 2016). Relationships between emotionality and concreteness have also been observed in corpus-based analyses, showing that positive and concrete words tend to occur in similar affective contexts, and that the words acquired earlier in the lexicon development are also those encountered in the higher positivity and concreteness contexts (Snefjella and Kuperman 2016). Importantly, as compared to concreteness, word imageability has been considered to better reflect the activity of mental imagery (Citron et al. 2014) and to be a stronger predictor of memory performance (Khanna and Cortese 2021; see also Cortese and Khanna 2022 for recent analyses including more words and context variables). In our study, we consistently found word imageability to interact with word emotionality in the two classical tasks used in episodic memory (i.e., free recall and recognition). If imageability per se is a critical factor to consider in episodic word memory, the present findings suggest that imageability is also important to consider in the effect of emotional words in episodic memory. To take this further, the memory advantage for high-imageability emotional words, especially for high-imageability positive words compared to high-imageability negative ones, suggests a specific processing system for this valence polarity. Positive high-imageability words were not only better recalled and recognized, they were also better discriminated and produced less false alarms than the other word conditions. Taken together, these data provide evidence for the fact that positive high-imageability words give rise to more accurate memories, which increases the ease with which they are retrieved in memory and discriminated in the recognition task. Previous studies have suggested that positive words benefit from a denser semantic network (Monnier and Syssau 2008) and are acquired earlier in human development (Snefjella and Kuperman 2016). High-imageable words also being semantically rich, high-imageable positive words are those that would accumulate the highest amount of semantic richness, hence increasing the ease with which accurate mental images can be created. In line with previous lexical decision task studies (Kanske and Kotz 2007; Yao et al. 2016), it could be assumed that high-imageability emotional words, especially positive ones, promote a mental imagery intervention in episodic memory. Mental imagery, which is involved in the memorization of high-imageability words (Paivio 1991), could therefore be a critical mechanism in the processing of emotional words (Holmes et al. 2008). Taken together, these results may help to clarify inconsistent findings on emotional words in the literature by highlighting the role of imageability in the processing of emotional words, and more precisely, positive words in episodic memory. Note that in our study, the recognition task always followed the recall task (see also Lohnas and Kahana 2013 for the same task order) because we were particularly interested in studying the combined effects of imageability and valence in the recall task which is well known to be sensitive to semantic factors (see Lau et al. 2018). Due to this constant task order, we cannot rule out the hypothesis that word recall has increased encoding thereby influencing the recognition performance. Future studies are necessary to clarify this issue. Finally, further experiments might also investigate the role of emotional context variables (Snefjella and Kuperman 2016; see also Cortese and Khanna 2022) on these effects in episodic memory. It is also noteworthy that an emotional word effect was found in the false alarms and *d*ʹ value for low-imageability words, indicating that low-imageability emotional words are less well discriminated than low-imageability neutral words. Since this effect was restricted to the false alarms and discrimination scores and not found on hit rates, it means that it was driven by the processes underlying the production of false alarms (see also Ballot et al. 2021; Cortese et al. 2004). Within dual process models (see Yonelinas 2002 for a review), false alarms are assumed to be underpinned by familiarity processes. Hence, the present data suggest that low-imageable emotional words are perceived as more familiar than low-imageable neutral words. Consequently, it would be more difficult to distinguish these familiar words as new words from learned words, thereby leading to an increase in the production of false alarms and difficulties in discriminating low-imageable emotional words from low-imageable neutral words.

To conclude, this study provides new findings showing a combined influence of word imageability and emotionality that should lead to a better understanding of the mechanisms underlying the episodic memory of emotional words. The finding that the effect of emotional words emerged only for highly-imageable words strongly suggests a critical role of mental imagery on the effects of emotional words in episodic memory. To go further in understanding the role of word imageability in emotional word episodic memory, future studies might also be designed to determine how and to what extent imageability is involved in the respective effects of emotional valence and arousal in episodic memory.
